# To Treat or Not to Treat: A Case of Simultaneous Discovery of Chronic Myelomonocytic Leukemia and Multiple Myeloma

**DOI:** 10.7759/cureus.17505

**Published:** 2021-08-27

**Authors:** Waleed Tariq Siddiqui, Mariam Farhan, Khanh Nguyen

**Affiliations:** 1 Internal Medicine, Griffin Hospital, Derby, USA; 2 Hematology and Oncology, Smilow Cancer Hospital Care Center, Derby, USA

**Keywords:** diagnosis of multiple myeloma, chronic myelomonocytic leukemia, hematological malignancy, monoclonal gammopathy, symptomatic anemia

## Abstract

The coexistence of multiple myeloma and chronic myelomonocytic leukemia in the same patient is a rare entity. Here we describe a case of an 80-year-old man who presented to our hospital with symptoms of dyspnea and found to have anemia and leukocytosis with peripheral monocytosis. Bone marrow biopsy, flow cytometry, and fluorescence in situ hybridization studies were consistent with a laboratory diagnosis of multiple myeloma and chronic myelomonocytic leukemia. Due to advanced age and multiple comorbidities, the patient was treated conservatively. At 26 months follow-up, the patient continues to do well.

## Introduction

Multiple myeloma (MM) is a plasma cell dyscrasia characterized by monoclonal proliferation of malignant plasma cells in the bone marrow, monoclonal protein in the serum, and organ dysfunction [1]. Chronic myelomonocytic leukemia (CMML) is a clonal malignancy-characterized abnormal proliferation of monocytes and myelocytes. This eventually leads to ineffective hematopoiesis of red blood cells and platelets in the bone marrow, resulting in anemia and bleeding. A diagnosis of CMML requires persistent monocytosis of greater than 1 x 10^9^/L in the peripheral blood, the absence of Philadelphia chromosome or the BCR-ABL fusion gene, less than 20% blasts in the blood or bone marrow, and dysplasia involving one or more myeloid lineages [2]. The coexistence of MM and CMML in the same patient is an extremely rare event and the management of this condition can prove to be challenging for physicians. Here we describe the clinical observation of a patient with simultaneous course of lymphoid and myeloid neoplasms.

## Case presentation

**Figure 1:**
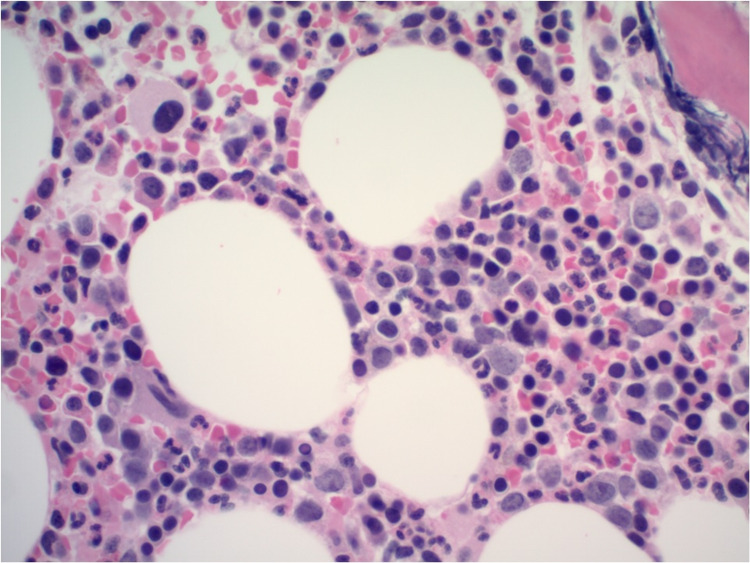
Hypercellular bone marrow for age (60% cellular) showing dysplastic megakaryocytes and an increase in plasma cells

**Figure 2:**
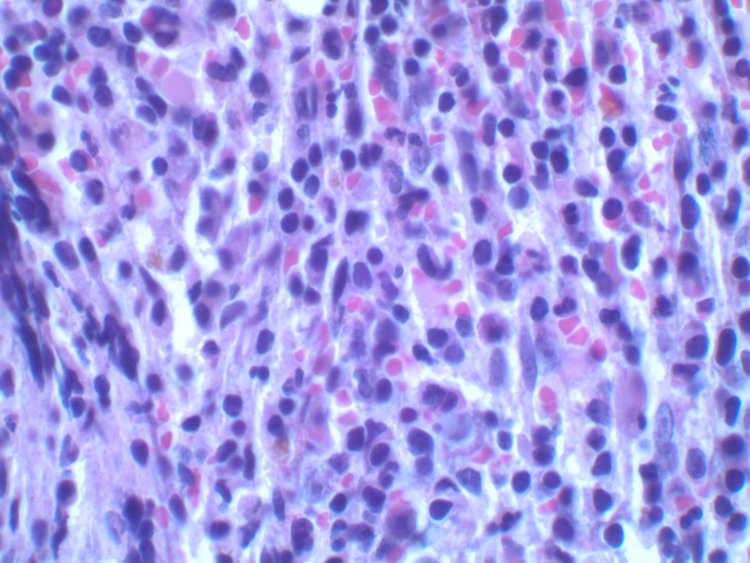
Bone marrow aspirate showing increased plasma cells, monocytosis, and dysplasia

**Figure 3:**
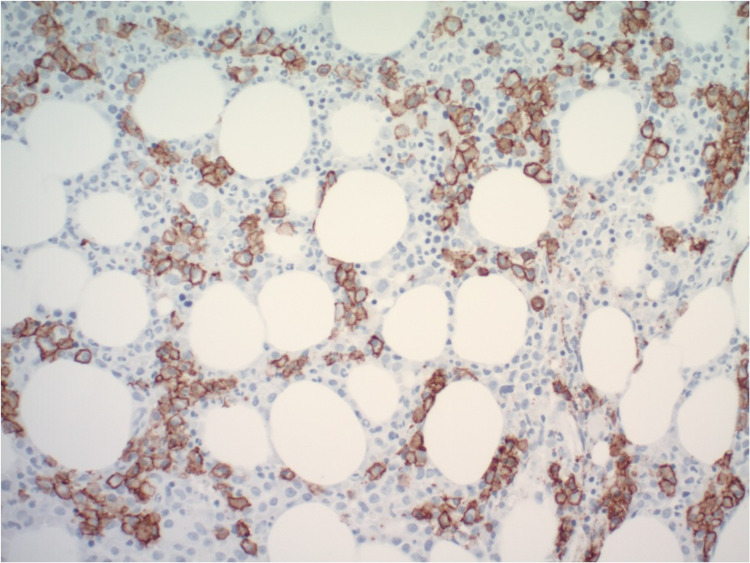
CD138-positive plasma cells are 30% of cellularity and show lambda restriction with lambda and kappa immunostains

**Figure 4:**
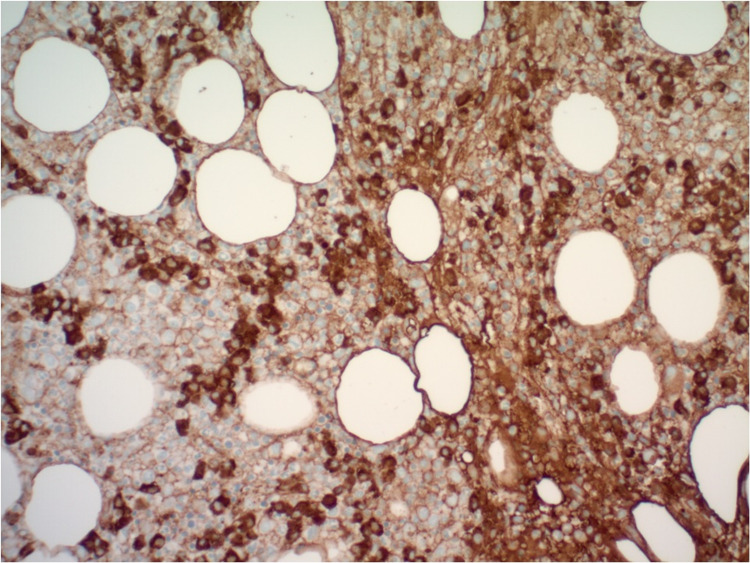
Plasma cells with dense lambda light chain

An 80-year-old male with extensive medical history including coronary artery disease, hypertension, type 2 diabetes mellitus, previous cerebrovascular accident without residual deficits, paroxysmal atrial flutter, and heart failure with preserved ejection fraction presented to the emergency department for evaluation of progressive dyspnea on exertion over the past two to three months with occasional night sweats. Laboratory investigations, including a complete blood count, showed a white blood cell count of 16,000 leukocytes with a differential count of 57.7% granulocytes, 6.9% lymphocytes, and 35.3% monocytes; hemoglobin of 7.0 g/dL; platelet count of 24,000/cumm; serum calcium level 9.1 mg/dL; and serum creatinine level of 1.0 mg/dL. Serum protein electrophoresis revealed marked elevation of serum IgA (2,010 mg/dL, normal 70-400 mg/dL) and beta 2 microglobulin (2.3 g/dL, normal 0.7-1.2 g/dL). The ratio of serum kappa/lambda free light chains was decreased to 0.17 (normal 0.26-1.65). A bone marrow aspirate revealed 60% hypercellularity (Figure 1) with plasma cells and myeloid dysplasia with monocytosis (Figure 2). A 500-cell count showed 4% blast equivalents (myeloblasts/monoblasts/promonocytes), 4% promyelocytes/myelocytes, 45% maturating granulocyte forms, 10% erythroid forms, 5% lymphocytes, 1% eosinophils, 9% plasma cells, 0% basophils/mast cells, and 22% monocytes. CD138-positive plasma cells were found singly and in clusters, and comprised approximately 30% of cellularity (Figure 3). The plasma cells were lambda restricted by kappa and lambda immunostains (Figure 4). CD34 highlighted <5% of cells. There were few CD117 highlighted scattered cells. Flow cytometric analysis showed mature immunophenotype monocytes that were increased to about 30% and expressed atypically, variable HLA-DR. There was also an abnormal CD10/CD13/CD16/CD11b myeloid maturation pattern. There was a normal number and phenotype myeloblasts with normal myeloid scatter by CD45/SSC. Chromosomal analysis was performed on direct and cultured unstimulated bone marrow cells. An abnormal pattern with a loss of Y chromosome was noted in all 20 metaphase cells analyzed. There were normal fluorescence in situ hybridization (FISH) patterns for the 5q, 7q, 8q (MYC), and 20q loci in blood leukocytes. Abnormal FISH results demonstrated four copies of 1q, three copies of 9q, and a large deletion of 13q in 30%, 6%, and 9% of enriched plasma cells (CD138+), respectively. These results were consistent with a diagnosis of CMML and plasma cell dyscrasia, i.e. MM [3,4]. On admission, he had received a unit of packed red blood cells for his anemia. However, hypercalcemia, renal failure, and bone lesions were not observed. Due to his advanced age and multiple comorbidities, it was decided to treat him conservatively with monthly follow-up.

## Discussion

The coexistence of MM and CMML is an extremely rare event. There are three case reports in the literature that have discussed this finding [3,4]. However, there have been case reports where patient being treated with an alkylating agent for MM later developed CMML. In a report by Ueki et al., in 37 patients with MM who were being treated with melphalan, three of those patients had later developed CMML as well [5]. A similar finding was observed by Natazuka et al., where a patient with MM treated with melphalan presented after 6.5 years with features of CMML [6]. Interestingly, our patient had never received any prior chemotherapy with an alkylating agent. The literature does describe some limited coexisting cases of plasma cell neoplasm with myelodysplastic syndromes. There are 24 case reports of coexisting MM and CML. Of these, there were only nine cases where both diseases expressed themselves simultaneously. In the remaining cases, MM preceded the diagnosis in eight cases and CML was diagnosed first in seven cases [7]. The co-occurrence of myeloproliferative and plasma cell disorder does raise the question whether if these two diseases share a common clonal origin. However, for now this question remains unanswered. Along with its diagnostic challenges, CMML also tends to have limited treatment options and a poor prognosis. For those who are not candidates for hematopoietic stem cell transplant, dysplastic CMML is managed with supportive care and azacitidine. Cytoreductive therapy is reserved for proliferative CMML [8].

Our patient has been treated conservatively with a monthly follow-up for more than two years. Each visit includes monitoring of complete blood count, chemistries, and serum protein electrophoresis (SPEP). Flow cytometry analysis is repeated intermittently. So far, he has been doing quite well. He does tend to have mild fatigue and bruising intermittently but remains largely asymptomatic. He has had no bleeding complications though has required intermittent blood transfusions for anemia. At 26-month follow-up, he remains clinically stable. His SPEP, serum immunoglobulins, and serum free light chains on most recent follow-up were all relatively stable.

## Conclusions

The co-occurrence of MM and CMML is an extremely rare entity that creates dilemmas in the management of these two separate malignancies. In prior literature, patients have been treated for either both malignancies or one of them. We present our experience with a patient who was diagnosed with these two malignancies simultaneously and treated conservatively. To our knowledge, this is the first such case where patient has been managed by observation alone with favorable outcome. Evaluation of further cases is required to determine the optimum management. Treatment with pharmacological agents should be considered on an individual basis.
